# Developing a COVID-19-focused mHealth system in a low-resource setting during the COVID-19 pandemic: challenges and opportunities

**DOI:** 10.3389/fdgth.2025.1543828

**Published:** 2025-06-19

**Authors:** Rosie Stenhouse, Wassie Gebbie Beshir, Demessie Girma, Gosaye Fida, Clara Calia, Godana Guto, Maria Klara Wolters

**Affiliations:** ^1^ School of Health in Social Science, University of Edinburgh, Edinburg, United Kingdom; ^2^ Faculty of Health Sciences, University of Maribor, Maribor, Slovenia; ^3^ Clinical Governance and Quality Improvement Unit, Adama Hospital Medical College, Adama, Ethiopia; ^4^ Oromia Tech plc, Adama, Ethiopia; ^5^ Scottish Collaboration for Public Health Research and Policy, University of Edinburgh, Edinburgh, United Kingdom; ^6^ Department of Internal Medicine, Adama Hospital Medical College, Adama, Ethiopia; ^7^ School of Informatics, University of Edinburgh, Edinburgh, United Kingdom

**Keywords:** Ethiopia, COVID-19, mHealth (mobile health), low-resource setting, pandemic, challenges

## Abstract

**Introduction:**

Approximately three-quarters of Ethiopia's population lives in rural areas, and access to healthcare is difficult with poor transport infrastructure and long travel times. Telemedicine has the potential to support healthcare access and minimise COVID-19 transmission through a reduced need to travel.

**Objectives:**

This Brief Research Report describes the analysis of qualitative data relating to the development of a mobile health (mHealth) system during the COVID-19 pandemic to support COVID-19 symptom management in the community in Oromia, Ethiopia.

**Methods:**

Data were collected from (1) meeting notes and WhatsApp group discussions, (2) a focus group with medical staff, and (3) an interview with a senior hospital leader. A framework method was used for the analysis.

**Results:**

Three themes were identified: (1) patient–physician relationship, (2) new ways of using everyday technology, and (3) infrastructure and digital access.

**Discussion:**

We discuss the challenges of developing an mHealth system during a pandemic alongside infrastructural challenges and the preparedness of medical staff and communities for the use of mHealth.

**Conclusions:**

There is a need for investment in information technology infrastructure and in access to digital networks, alongside a need to improve the digital and health literacy of populations for the successful implementation of a patient-facing mHealth system. Thus, whilst the policy aspirations are admirable, the potential for technological innovation is great, and the clinicians can see the benefit of using technologies to provide care to those who cannot reach clinics, there is a gap between what is possible given the current reality of infrastructure and patient preparedness and the requirements for a successful telemedicine intervention.

## Introduction

Telemedicine has the potential to support high-quality care in situations where transport infrastructure is poor and travel times are long (1). This is the case in Ethiopia, a country in eastern sub-Saharan Africa. Approximately three-quarters (77%) of Ethiopia's population lives in rural areas (2), yet public hospitals in Ethiopia's three-tier healthcare system are primarily situated in urban areas. Therefore, for rural populations, accessing hospitals requires long journeys by car or public transport. Telemedicine and telehealth differ in their scope, and we use these terms in line with those used by the authors whose papers we cite.

Telemedicine became particularly important during the COVID-19 pandemic, when measures such as social distancing, testing, contact tracing, and quarantine were used to contain viral transmission (3, 4). Since telemedicine supports symptom management for patients at home (5), it allowed those with COVID-19 symptoms to remain at home instead of travelling on public transport with the concurrent risk of viral transmission.

Internet and cell-phone penetration in Ethiopia are 19.4% and 60.4%, respectively (6). Prior work has shown that telehealth services, in particular phone-based services, are viable in Ethiopia. Gebremariam et al. (7) report that for parents in Tigray, Ethiopia, mobile phone use was well-integrated into everyday life. Jemere et al. (8) found high levels of access to mobile phones and willingness to receive mobile phone-based health services in a survey of 423 diabetes patients in Northwest Ethiopia. Access and willingness were significantly associated with the educational level of participants and other factors, including the distance to the clinic. Assaye and Shimie (9) report that two-thirds of the clinicians who participated in their survey of telemedicine use in Ethiopia were already using one or more forms of telemedicine in their practice. Yet, even when clinicians and healthcare workers view telemedicine positively, IT infrastructure, IT support, and cost are frequently cited as issues (10–13). In addition, clinicians and healthcare workers may feel anxious about technology and worry about its effect on the patient–physician relationship (14, 15). Training also plays a substantial role. Shiferaw et al. (16) found that if clinicians are confident that they are able to use telemedicine well and without much additional effort, they are more likely to adopt it. Social influence may also play a considerable role (16).

Health literacy is broadly defined as the ability of individuals and communities to access, understand, and apply health-related information to prevent and manage health conditions (17). In their evidence synthesis of 16 studies examining health literacy in Ethiopian populations, Adamu et al. (18) found that health literacy levels were regarded as low or insufficient. However, the populations included in the primary studies were limited to students and patients, leading the authors to conclude that the evidence was limited in its scope. Studies that examined Ethiopians' health literacy with respect to COVID-19 confirm this assessment. In an online survey of 929 participants across Ethiopia, Kebede et al. (19) found a range of myths and false assurances of safety related to COVID-19, with the highest information needs evident in large cities. Kebede et al. (19) acknowledge that their 929 online survey participants were generally educated with access to the Internet, and their views of community behaviours and knowledge were a proxy. An interview study of 247 visitors at a University Medical Centre in Oromia during the early months of the pandemic showed that only 41.9% of respondents had sufficient knowledge about COVID-19, and that younger, employed visitors with more knowledge and better self-efficacy were more likely to engage in effective preventative practices (20).

There was therefore clear evidence for the potential feasibility of a mobile health (mHealth) approach to managing COVID-19 infections. This project developed from conversations between DG, an Ethiopian technology innovator and GJ, a physician running COVID-19 services in an Oromian University Hospital. The purpose of the project was to develop and test a prototype of an mHealth system that would support the work of clinicians in treating patients with COVID-19 symptoms, reducing viral transmission by enabling people to remain in their communities. The mHealth system consisted of an application and a clinical dashboard, enabling individuals in the community to flag their symptom status to medics based in the urban hospitals who used the clinical dashboard to monitor patients’ health status and provide advice, including requesting that patients attend hospital or clinics.

This article presents an analysis of the qualitative process data generated during the project aimed at the development and pilot testing of the mHealth system during the first 2 years of the pandemic (2020/21). The question driving the analysis in this article is “What is needed to support the development and implementation of a patient-facing mHealth approach to pandemic management in Ethiopia?” Our data covers the entire development process of the system, from initial ideation to trial deployment of a dashboard. The qualitative data documents how clinical user stakeholders were involved in the entire process and key interactions between technical and clinical stakeholders.

## Methods

### Design

A qualitative approach is used to enable an understanding of what is needed to support the development and implementation of the mHealth system.

### Setting

The setting was a mix of rural and urban communities in Oromia, Ethiopia. During the mHealth system development, we engaged with medical staff from a teaching hospital and a smaller rural hospital in the region.

### Sample

Data for this article were generated from the research team, six medics in the teaching hospital who had been engaging with mHealth development and training, and a senior leader in the teaching hospital.

### Data generation

Qualitative data comprised documentary text in the form of meeting notes and WhatsApp messages, and verbal data from one interview and one focus group. The research team convened in a WhatsApp group, used for ongoing discussion about challenges and potential solutions and for monthly synchronous team meetings. The team meetings facilitated real-time discussion of project progress and enabled the team to make key decisions on application development and setting up the structures for the implementation of the pilot. The meetings lasted between 1 and 1.5 h. WhatsApp was chosen as it was easily accessed by all team members, provided encryption to ensure a measure of data security, required the least amount of mobile data, which was important for the inclusion of team members situated in Ethiopia, and allowed the team to easily link synchronous and asynchronous discussions. Attempts to use MS Teams or Zoom for synchronous meetings resulted in problematic connectivity. RS kept notes of the discussions at these monthly meetings. The WhatsApp data and related notes enabled us to capture the process of developing the mHealth system, including the engagements with wider stakeholders in relation to development and setting up the pilot of the system.

To capture the experience and expectations of the staff in the hospital who were expected to engage with the mHealth system, and who had been involved in training sessions run by the local system developer, DG, we undertook a focus group with medical staff (*n* = 6). Topics covered in the focus group were

• confidence with computers;
• clinical dashboard training;
• usability of the dashboard;
• using the system to manage patients;
• clinical management and technology;
• talking to patients about the mHealth system.

The focus group was co-facilitated by RS on MS Teams and WG *in situ* with the participants. It was recorded on MS Teams and transcribed verbatim by RS; WG checked and amended the transcript for accuracy. In addition, a virtual interview using MS Teams was undertaken with a senior leader in the Hospital by RS and covered the following topics:

• the experience of partnership working;
• opportunities and challenges;
• supporting staff engagement;
• medical/healthcare staff education on technology.

The interview was recorded, and detailed notes were made for inclusion in the analysis.

### Data analysis

An abductive approach (21), using a framework method (22), was used, enabling analysis of data from a range of sources. A framework containing *a priori* codes was developed from the Ethiopian Ministry of Health's (23) policy guidance on implementing telehealth. Transcripts were initially coded independently of the framework to identify aspects of the data that went beyond the framework. Initial coding was undertaken by RS and checked with WG and MW. The framework was then used to organise the codes and expanded as per Table 1.

**Table 1:** Analytic framework developed from the pre-requisites for a telehealth service in the Telehealth Guideline: Practical Tips of the Ethiopian Ministry of Health (23).

MoH telehealth guideline items/additional categories from data	Analytic codes	Thematic contribution
The ability to communicate under a variety of conditions	No data	
Have an understanding of the scope of service being provided via telehealth	No data	
Well-versed IT professionals’ presence	No data	
A short orientation to the technology, navigation, and telehealth environment	Impact of orientation to mHealth dashboard	New ways of using everyday technology
Operational protocols and procedures, such as scheduling, reserving rooms, and timeliness	Responsiveness to patients	Patient–physician relationship
Professional telemedicine guidelines and functional internet	Infrastructure challenges	Infrastructure and digital access
	System guidance for staff	Infrastructure and digital access
Trained clinical teams on the use of equipment and other technicalities	Computer use academic only	New ways of using everyday technology
	Lack of familiarity with apps/telehealth	New ways of using everyday technology
	Motivated/enthusiastic to develop telehealth skills	New ways of using everyday technology
Government policy as driver	Aspiration to meet policy	New ways of using everyday technology
	IT as solution to distance	New ways of using everyday technology
Digital access and literacy of population	Poor digital access	Infrastructure and digital access
	Digital literacy	Infrastructure and digital access
Impact on clinical care	Functionality of app	Patient–physician relationship
	Patient safety	Patient–physician relationship
Ethical considerations	Patient privacy	Patient–physician relationship
Disseminating telehealth application	Medical staffs’ role in disseminating telehealth	Patient–physician relationship
	Patient scepticism	Patient–physician relationship

### Ethical considerations

Ethical approval was obtained from the School of Health in Social Science, University of Edinburgh and Adama Hospital Medical College. Participation in the focus group and interview was voluntary and informed consent was recorded. Participants were allocated and identified by participant number to preserve anonymity. Due to the single interview with the senior manager, the participants in the interview and focus group are not differentiated by role and are simply referred to as P1, P2, etc. All are medically trained and working within the research site.

## Results

Three key themes were identified in the data: (1) patient–physician relationship, (2) new ways of using everyday technology, and (3) infrastructure and systems.

### Patient–physician relationship

#### Patient safety

This theme captured the central concerns of the physician participants in relation to the potential impact of using the mHealth system for remote clinical care and symptom monitoring. The clinicians identified the importance of being able to examine patients to inform diagnostic and treatment decisions. The ability to physically examine and interact with patients is in stark contrast to the operation of the mHealth system, which would require them to rely on patient reporting of symptoms, and consequently rest on patients’ health literacy and decisions about what to tell the physician and what, to them, was not important.

“But problems may arise when it comes to patient privacy …. The application requires some sort of information including the phone number and for the sake of their own privacy the patients may decline to give this information and when it comes to their manifestations [symptoms] they may not give us full information.” (P3)

“The other thing is that would be symptom reporting. What we are accustomed to is taking patient history, doing physical examination, doing something in the lab. So like this thing's [the mHealth system] a new thing so we try to gather information about our patients just by their reporting. So when we talk about COVID there are also other comorbid conditions in COVID patients. So we may need those things, I mean for patients it may seem a simple thing but when we see them it may be an important finding for ourselves as physicians.” (P4)

This was particularly the case where patients might have co-morbidities that would impact the COVID-19 illness trajectory and inform decisions about where or how to care for a patient with symptoms. Thus, the ability to interact in real time with patients, probing their self-reports, and undertaking physical examination where appropriate, was a key aspect of physicians keeping patients safe. To maintain patient safety, the physicians suggested a shift to where in the patient journey the system might be used, suggesting that it be focused for follow-up of patients who had been inpatients and were discharged back into the community.

“It is not sufficient to, based on self-report, give some advice about the condition. But for COVID-19 patients who are investigated, discharged … for daily reporting of their symptom, it's more applicable for those patients … For new patient with no medical history and no investigation, as a new patient to start with this application it seems difficult for us.” (P6)

Despite these concerns, the physician participants demonstrated enthusiasm for the use of mHealth. They engaged with the system developer during the training sessions, where they received a demonstration and training on the system, providing feedback on how the clinician dashboard might be improved to increase patient safety.

#### Physicians as promoters of the system

Physicians during training also made suggestions about the best routes to engage patients with the system. They identified that they were trusted by patients and, therefore, their support would be crucial to the rollout of the system into local communities.

“Not very easy to get into community and interact with people to get engagement. Doctors best to get patients to engage with the app. Need to exploit connections between different areas of the service. Relationship between lower hospitals and treatment centres needs to be understood.” (Meeting notes 7 July 2021)

“You know these are busy guys and that is why they have not got the time to promote the app to individuals when they are working in the critical care and so on.” (P8)

However, whilst they might be the most trusted healthcare professional, the participants also acknowledged their limited capacity to promote the system due to time pressures created by workload. Interestingly, the physicians in the more rural group suggested that the route to engage with community members regarding the mHealth system would be through engagement with health workers in local health centres.

“Discussion with [rural area] medics indicates would be more successful for roll out of app into community via medics and healthcare professionals capitalising on the trust the community has in these people. Potential to use the network of local hospitals and health centres for this.” (Meeting notes 28 July 2021)

This may point to the need to develop contextually dependent routes into communities, or that the rural situation of these physicians meant that they had more connection with these centres and, therefore, were more likely to consider them a useful way to engage communities with the system.

### New ways of using everyday technology

The data demonstrated that whilst physicians used computers regularly in their work, this was related to education rather than clinical aspects of their work.

“We are using computers on a daily basis especially for academic purpose and so we can use … the only new thing is that maybe being familiar with these apps and how to communicate with our patients and refer different patients.” (P1)

“Actually we don't have this network [referring to lack of IT network within hospital setting], it [clinical information] comes through as paper … we are trying in this school to adapt with other organisations so that every computer will be connected either with LAN or with internet so that we can receive results from the labs via the PC and so on. But currently we are paper based workers here for some time.” (P8)

Despite this, the physicians were enthusiastic about the use of technology in their clinical practice and were confident they would be able to use the mHealth system.

Whilst it was acknowledged that increasing numbers of Ethiopians possessed smartphones, they were understood to use these for engaging with social media rather than engaging with learning or health.

“Technology has potential but there are issues with cultural attitudes towards it. Many people have got smartphones but don't use them to develop knowledge, just to go on social media.” (P7)

This issue of how technology was currently used by the population was spoken about by the local members of the research team, the physicians, and the senior leader, and was perceived to be a potential barrier to engagement with the mHealth system.

### Infrastructure and digital access

The data illuminated the challenges posed by the infrastructure currently available to the health system in Ethiopia, including regular electricity and internet outages. Such outages interfered with the reliability of any digital system. Whilst the internet outages in the clinical setting were problematic, an additional problem existed in relation to a lack of publicly available internet and the need for people in the community to use their data function to access the internet.

“The problem is specially wifi and connection most patients have not that … In the rural area they [patients] have no mobile [phone] to use this application they have no wifi …. as well as electricity interruptions, this is my fear to continue with this app and in the future if these problems are fixed I think we can proceed.” (P6)

“Most people in the community use data as Wifi is only in government buildings or private service providers.” (P7)

The reliance on data implies a potential financial burden for community members trying to access the mHealth system, and consequent reliability issues if people run out of data.

In addition, the technology systems ordinarily available in clinical settings were not necessarily the most up-to-date, creating challenges for a cloud-based mHealth system. The local developer of the mHealth system had developed it to work on Windows 10; however, many of the local computers were running Windows 7, leading to a need to buy licenses to install Windows 10. This led to the following exchange in our WhatsApp group.

DG: The App doesn't really make out of the norm requirements considering that the minimum Android platform required is over 7 years old (Android Version 5.0, 25 June 2014) and for the Dashboard the minimum Windows platform required is over 6 years old (Windows 10, July 2015)….

MW: If we have a good idea of the kinds of IT actually available in hospitals and health centres, we can then map the Fayyaa Ko requirements against what people on the ground have access to….

WG: Yes Windows in the hospital do not require such updates for use of MSexcel and word, and powerpoint, so this is normal here, you should expect this problem [lack of access to Windows 10]

This exchange surfaces the challenges created by what seemed to be a reasonable assumption about the software in current use in the clinical areas. This occurred even where the technology developer is locally based, and despite ongoing interaction with the technology end users and the inclusion of medics on our research team.

## Discussion

This study focused on understanding what is needed to support the development and implementation of a patient-facing mHealth approach to pandemics in Ethiopia. While most of the literature reports existing investigations and preliminary research with stakeholders, this study presents an inside view of the development process of an application under tight time and resource constraints. The findings highlight a tension between the physicians’ perceptions of the opportunities provided by the mHealth application, the potential risks posed to patient safety, and the technology required for a robust and secure cloud-based solution. The risks identified by clinicians covered a number of aspects, including the inability to physically examine the patient, concerns about infrastructural issues such as internet and electricity availability, and the issues raised by the economic status of the potential patients and how this would impact their ability to consistently access a mobile phone or mobile data. Perhaps in addition to these issues, the novelty of the COVID-19 virus and the limited understanding of the impact on other comorbid disease states that was available initially during the pandemic created a further layer of anxiety about the distance between physician and patient.

Smith et al. (5) identify the suitability of telehealth for the management of communicable diseases. However, they propose that this occurs most successfully where the clinical workforce has the skills and familiarity through regular use of telehealth systems. Thus, readiness to use telemedicine is necessary for successful implementation. Seboka et al. (24) found low levels of readiness (65%) to use telemedicine in their cohort of 423 physicians and nurses in teaching hospitals in Ethiopia, contrasting this with the 40% of nurses in a US study who had high levels of readiness. Similarly to Nizeyimana et al. (25), the key barriers identified in our study are infrastructural and resource-related challenges. Unreliable electricity supply, WiFi/data costs and availability, and access to the required digital hardware, such as smartphones, all impact the possibility of developing patient-facing telehealth systems for regular patient management. Such organisational readiness is a key aspect of a successful telehealth implementation (25). If telemedicine readiness is partly facilitated by regular telemedicine use, and regular telemedicine use is constitutive of the successful deployment of such systems in emergencies, this poses challenges for low-resource countries. This means that the adoption of such systems in the face of emergencies such as the COVID-19 pandemic is less likely to be successful than in high-resource countries where these are better integrated into the healthcare system.

The development of an mHealth system in the midst of an unfolding pandemic meant that there was limited time, and in-person access to patients, especially those living in rural areas, was not possible. The physicians’ concerns about the best point in a patient's journey for the mHealth system to be used highlight the importance of taking a user-centred approach from the beginning of a project (26). In particular, to the best of our knowledge, there was no prior work on patient readiness to use smartphones for infectious disease management in Ethiopia. Most of the previous research on the acceptability of mobile phones considered both smartphones and cellphones that only offer access to calls, texts, and limited applications such as WhatsApp (7, 27). Within the context of implementing telehealth during emergencies, this points to the need for investment in telehealth infrastructure, which can then be built upon in the context of emergencies. It also highlights the need to conduct regular investigations into the preparedness of people to use their smartphones for medical purposes, and the spread of smartphones in the population, particularly in areas with fewer financial and infrastructure resources.

In addition to the infrastructural and contextual challenges in the development of the mHealth system, the digital and health literacy of the population was likely to impact patients’ ability to use the system and the system's ability to support patient safety. The purpose of this mHealth system was to provide remote monitoring to people with COVID-19 symptoms in rural areas. However, these populations are likely to experience poverty and food and clean water insecurity, all of which are associated with lower educational engagement (26) and lower levels of health literacy (17, 17, 29) in comparison to their urban counterparts. Whilst the digital and health literacy of the population did not appear in the pre-requisites in the telehealth document from which the framework for the analysis was developed, it is clear that this was a concern of the physicians. Health literacy is improved by contextually appropriate and easily understood health communication (29), yet during the COVID-19 pandemic, there was an overwhelming amount of information and misinformation in circulation (30). In a survey of 6,007 participants from high-risk groups, such as healthcare workers and public transport drivers in Addis Ababa, Defar et al. (31) found that approximately half had knowledge of the COVID-19 modes of transmission and 60% of prevention measures. In their Ethiopia-wide study, Kebede et al. (19) identified several widespread myths and false assurances, and, on average, 59% of respondents per region voiced further information needs. These findings highlight the challenges in supporting health literacy during the COVID-19 pandemic where the evolving evidence-base left the door open for a range of misinformation, and illuminate the challenges of developing and implementing an mHealth system that requires population health literacy.

## Conclusion

A successful implementation of a patient-facing telehealth system needs considerable government investment in the electricity and internet infrastructure in hospitals/clinical settings and urban and rural settlements. In addition, appropriate IT devices across rural and urban communities are required. There is also a need to improve patient preparedness through digital skills and health literacy programmes to optimise patient safety and good clinical decision-making. Thus, whilst the policy aspirations are admirable, the potential for technological innovation is great, and the clinicians can see the benefit of using technologies to provide care to those who cannot reach clinics, there is a gap between what is possible given the current reality of infrastructure and patient preparedness and the requirements for a successful telemedicine intervention.

## Data Availability

The pseudonymised data supporting the conclusions of this article will be made available by the corresponding author, upon reasonable request.

## References

[1] Anawade PA Sharma D Gahane S. A comprehensive review on exploring the impact of telemedicine on healthcare accessibility. Cureus. (2024) 16(3):e55996. 10.7759/cureus.55996 38618307 PMC11009553

[2] Trading Economics. Ethiopia—Rural Population. (2024). Available at: https://tradingeconomics.com/ethiopia/rural-population-percent-of-total-population-wb-data.html (Accessed June 10, 2025).

[3] FMOH. National Comprehensive COVID-19 Management Handbook. Addis Ababa: Federal Ministry of Health (2020). Available at: http://www.dataverse.nipn.ephi.gov.et/bitstream/handle/123456789/749/COVID%2019%20Handbook%20for%20health%20professionals%20FMOH%202020.pdf?sequence=1&isAllowed=y

[4] Shigute Z Mebratie AD Alemu G Bedi A. Containing the spread of COVID-19 in Ethiopia. J Glob Health. (2020) 10(1):010369. 10.7189/jogh.10.010369 32566160 PMC7296219

[5] Smith AC Thomas E Snoswell CL Haydon H Mehtrotra A Clemensen J Telehealth for global emergencies: implications for coronavirus disease 2019 (COVID-19). J Telemed Telecare. (2020) 26(5):309–13. 10.1177/1357633X20916567 32196391 PMC7140977

[6] Kemp S. Digital 2024: Ethiopia (2024). (Datareportal accessed at Digital 2024: Ethiopia—DataReportal—Global Digital Insights on 18/10/2024). Available at: https://datareportal.com/reports/digital-2024-ethiopia (Accessed June 10, 2025).

[7] Gebremariam KT Zelenko O Hadush Z Mulugeta A Gallegos D. Could mobile phone text messages be used for infant feeding education in Ethiopia? A formative qualitative study. Health Inform J. (2020) 26(4):2614–24. 10.1177/1460458220911779 32308097

[8] Jemere AT Yeneneh YE Tilahun B Fritz F Alemu S Kebede M. Access to mobile phone and willingness to receive mHealth services among patients with diabetes in northwest Ethiopia: a cross-sectional study. BMJ Open. (2019) 9(1):e021766. 10.1136/bmjopen-2018-021766 30679284 PMC6347931

[9] Assaye BT Shimie AW. Telemedicine use during COVID-19 pandemics and associated factors among health professionals working in health facilities at resource-limited setting 2021. Inform Med Unlocked. (2022) 33:101085. 10.1016/j.imu.2022.101085 36105540 PMC9462923

[10] Assaye BT Jemere AT Nigatu AM. Knowledge and awareness of health professionals towards telemedicine services in northwest, Ethiopia. Digit Health. (2022) 8:20552076221143250. 10.1177/20552076221143250 36478985 PMC9720804

[11] Medhanyie AA Little A Yebyo H Spigt M Tadesse K Blanco R Health workers’ experiences, barriers, preferences and motivating factors in using mHealth forms in Ethiopia. Hum Resour Health. (2015) 13(1):2. 10.1186/1478-4491-13-2 25588973 PMC4325949

[12] Sagaro GG Battineni G Amenta F. Barriers to sustainable telemedicine implementation in Ethiopia: a systematic review. Telemed Rep. (2020) 1(1):8–15. 10.1089/tmr.2020.0002 35722252 PMC8812291

[13] Manyazewal T Woldeamanuel Y Blumberg HM Fekadu A Marconi VC. The potential use of digital health technologies in the African context: a systematic review of evidence from Ethiopia. NPJ Digit Med. (2021) 4(1):125. 10.1038/s41746-021-00487-4 34404895 PMC8371011

[14] Ahmed MH Awol SM Kanfe SG Hailegebreal S Debele GR Dube GN Willingness to use telemedicine during COVID-19 among health professionals in a low income country. Inform Med Unlocked. (2021) 27:100783. 10.1016/j.imu.2021.100783 34778509 PMC8571100

[15] Xue Y Liang H Mbarika V Hauser R Schwager P Kassa Getahun M. Investigating the resistance to telemedicine in Ethiopia. Int J Med Inf. (2015) 84(8):537–47. 10.1016/j.ijmedinf.2015.04.005 25991059

[16] Shiferaw KB Mengiste SA Gullslett MK Zeleke AA Tilahun B Tebeje T Healthcare providers’ acceptance of telemedicine and preference of modalities during COVID-19 pandemics in a low-resource setting: an extended UTAUT model. PLoS One. (2021) 16(4):e0250220. 10.1371/journal.pone.0250220 33886625 PMC8061916

[17] World Health Organisation (WHO). Health Literacy: The Mandate for Health Literacy. Geneva: World Health Organisation (2024). Available at: https://www.who.int/teams/health-promotion/enhanced-wellbeing/ninth-global-conference/health-literacy (Accessed July 9, 2024).

[18] Adamu AA Godesso A Birhanu Z. Health literacy in Ethiopia: evidence synthesis and implications. J Multidiscip Healthc. (2023) 16:4071–89. 10.2147/JMDH.S440406 38116303 PMC10729771

[19] Kebede Y Birhanu Z Fufa D Yitayih Y Abafita J Belay A Myths, beliefs, and perceptions about COVID-19 in Ethiopia: a need to address information gaps and enable combating efforts. PLoS One. (2020) 15:1–18. 10.1371/journal.pone.0243024 PMC7703946 33253268

[20] Kebede Y Yitayih Y Birhanu Z Mekonen S Ambelu A. Knowledge, perceptions and preventive practices towards COVID-19 early in the outbreak among Imma university medical center visitors, southwest Ethiopia. PLoS One. (2020) 15(5):e0233744. 10.1371/journal.pone.0233744 32437432 PMC7241810

[21] Blaikie N. Designing Social Research: The Logic of Anticipation. 3rd edn. Newark: Polity Press (2018).

[22] Gale NK Heath G Cameron E Rashid S Redwood S. Using the framework method for the analysis of qualitative data in multi-disciplinary health research. BMC Med Res Methodol. (2013) 13:1–8. 10.1186/1471-2288-13-117 24047204 PMC3848812

[23] Ministry of Health. Telehealth Implementation Guideline Practical Tips. Addis Ababa: Federal Ministry of Health (2020). 10.13140/RG.2.2.31548.51847

[24] Seboka BT Yilma TM Birhanu AY. Awareness and readiness to use telemonitoring to support diabetes care among care providers at teaching hospitals in Ethiopia: an institution-based crosssectional study. BMJ Open. (2021) 11:e050812. 10.1136/bmjopen-2021-050812 34716162 PMC8559102

[25] Nizeyimana E Joseph C Louw QA. Organizational readiness and rehabilitation professionals’ views on integrating telerehabilitation into service delivery and students’ clinical training: a qualitative study. Digit Health. (2023) 9:1–12. 10.1177/20552076231212314 PMC10631339 38025095

[26] Carey N Abathun E Maguire R Wodaje Y Royce C Ayers N. Co-design and prototype development of the ‘Ayzot App’: a mobile phone based remote monitoring system for palliative care. Palliat Med. (2023) 37(5):771–81. 10.1177/02692163231162408 37002562 PMC10227095

[27] Kassa ZY Tenaw Z Astatkie A Siyoum M Bekele G Taye K. Mobile phone based strategies for preconception education in rural Africa. Ann Glob Health. (2019) 85(1):101. 10.5334/aogh.2566 31298825 PMC6634418

[28] Devonald M Jones N Yadete W. Addressing educational attainment inequities in rural Ethiopia: leave no adolescent behind. Dev Policy Rev. (2021) 39:740–56. 10.1111/dpr.12523

[29] Fitzpatrick PJ. Improving health literacy using the power of digital communications to achieve better health outcomes for patients and practitioners. Front Digit Health. (2023) 5:1264780. 10.3389/fdgth.2023.1264780 38046643 PMC10693297

[30] Wei CR Lang’at GC. Health and digital literacy: essential tools for Ethiopians during COVID-19. Ann Med Res Public Health. (2023) 1(2):1–4.

[31] Defar A Molla G Abdella S Tessema M Ahmed M Tadele A Knowledge, practice and associated factors towards the prevention of COVID-19 among high-risk groups: a cross-sectional study in Addis Ababa, Ethiopia. PLoS One. (2021) 16(3):e0248420. 10.1371/journal.pone.0248420 33705480 PMC7951807

